# The MYB/miR-130a/NDRG2 axis modulates tumor proliferation and metastatic potential in salivary adenoid cystic carcinoma

**DOI:** 10.1038/s41419-018-0966-2

**Published:** 2018-09-11

**Authors:** Yu Wang, Chun-ye Zhang, Rong-hui Xia, Jing Han, Bao Sun, Shu-yang Sun, Jiang Li

**Affiliations:** Department of Oral Pathology, Shanghai Ninth People’s Hospital, College of Stomatology, Shanghai Jiao Tong University School of Medicine, National Clinical Research Center for Oral Diseases, Shanghai Key Laboratory of Stomatology & Shanghai Research Institute of Stomatology, Shanghai, China

## Abstract

Increasing evidence has emerged to suggest that N-myc downstream-regulated gene 2 (NDRG2) dysregulation participates in a number of tumor biological processes. However, the role of NDRG2 and miRNA-mediated NDRG2 regulation in salivary adenoid cystic carcinoma (SACC) progression remain unknown. Here, we determined that SACC tissues exhibited decreased level of NDRG2, which was associated with poorer rates of overall survival and distant metastasis-free survival. Silencing NDRG2 promoted SACC cell proliferation and metastasis both in vitro and in vivo. MiRNAs have been reported as vital regulators of NDRG2 expression. Based on micronome sequencing of three paired samples of SACC and normal salivary gland tissue and on an online database analysis, miR-130a was identified as a candidate miRNA that potentially regulates NDRG2. We demonstrated that the expression level of NDRG2 was dramatically reduced by exogenous miR-130a. Moreover, a luciferase assay further validated that miR-130a could degrade NDRG2 mRNA by targeting sites in the NDRG2 3′UTR. A rescue experiment suggested that NDRG2 expression could reverse the miR-130a-mediated promotion of cell proliferation and invasion. The expression of miR-130a has been reported to be regulated by certain transcription factors. In the preset study, we verified that the transcription factor MYB acted as the critical driver in SACC-upregulated miR-130a expression directly and induced NDRG2 downregulation in SACC tissues. Additionally, MYB/miR-130a activated the STAT3 and AKT pathways by downregulating NDRG2. These observations suggest that the MYB/miR-130a/NDRG2 axis, which modulates proliferation and metastasis in SACC, provides promising targets for the treatment of SACC.

## Introduction

SACC is the most common malignancy of the salivary gland, accounting for 30.42% of all salivary malignant tumors in the Chinese population^1^. The unfavorable prognosis and inferior overall survival rate of SACC are the essential consequence of its aggressive and unique characteristics; SACC is associated with a high rate of relapse, perineural invasion in the early phase, and late distal metastasis, particularly in the lungs^2^. Studies of the mutational landscape have found that the recurrent MYB–NFIB translocation, resulting in a highly expressed MYB gene, is the main genomic hallmark of SACC^3^. Nevertheless, the molecular mechanism of how the critical oncogenic driver MYB influences tumor progression has not yet been fully elucidated^4–6^.

NDRG2, which is implicated in nervous system diseases and human carcinoma, has recently been reported as a candidate tumor suppressor gene^7^. It has been revealed to interact with the ubiquitously protein PRA1 which can modulate vesicular trafficking, lipid transport and cell migration^8^. The lack of NDRG2 in T cell leukemia/lymphoma and other malignancies enhances activation of PI3K-AKT and NF-KB signaling through PTEN and NIK phosphorylation^9^. We believe that the clinical significance and exact biological function of NDRG2 in SACC deserve much investigation.

A panel of miRNAs has been identified to involve in human cancers, but very limited studies on their role in SACC has been conducted^10^. Increasing evidence demonstrated that miR-130a is downregulated and could act as a tumor suppressor in certain cancer types, including breast cancer^11^, gastric carcinoma^12^ and prostate carcinoma^13^. However, miR-130a has also been found to exhibit oncogenic characteristics in cervical cancer^14^, osteosarcoma and ovarian carcinoma^15^. The dual effects of miR-130a may result from the tissue-specificity and the distinct cellular environment. However, whether miR-130a contributes to SACC progression or not still remains unknown.

In our present study, the biological role of NDRG2, miR-130a and MYB was determined in the development and progression of SACC. We verified that NDRG2 is downregulated in most SACC samples and the correlation between downregulation of NDRG2 and the poor prognosis of SACC patients is significant. We also demonstrated that NDRG2 is a direct target of miR-130a, an oncomiR in SACC which can be activated by the transcription factor MYB. Furthermore, NDRG2 can reverse the oncogenic effects of miR-130a and repress the STAT3 and AKT signaling pathways, which are upregulated by this miRNA. Overall, these data strongly suggest that the MYB/miR-130a/NDRG2 axis, which modulates SACC tumorigenesis and metastasis, may provide promising targets for the treatment of SACC.

## Results

### NDRG2 is downregulated in SACC tissue samples, and a low NDRG2 expression level is associated with distant metastasis and poorer survival

**Fig. 1:**
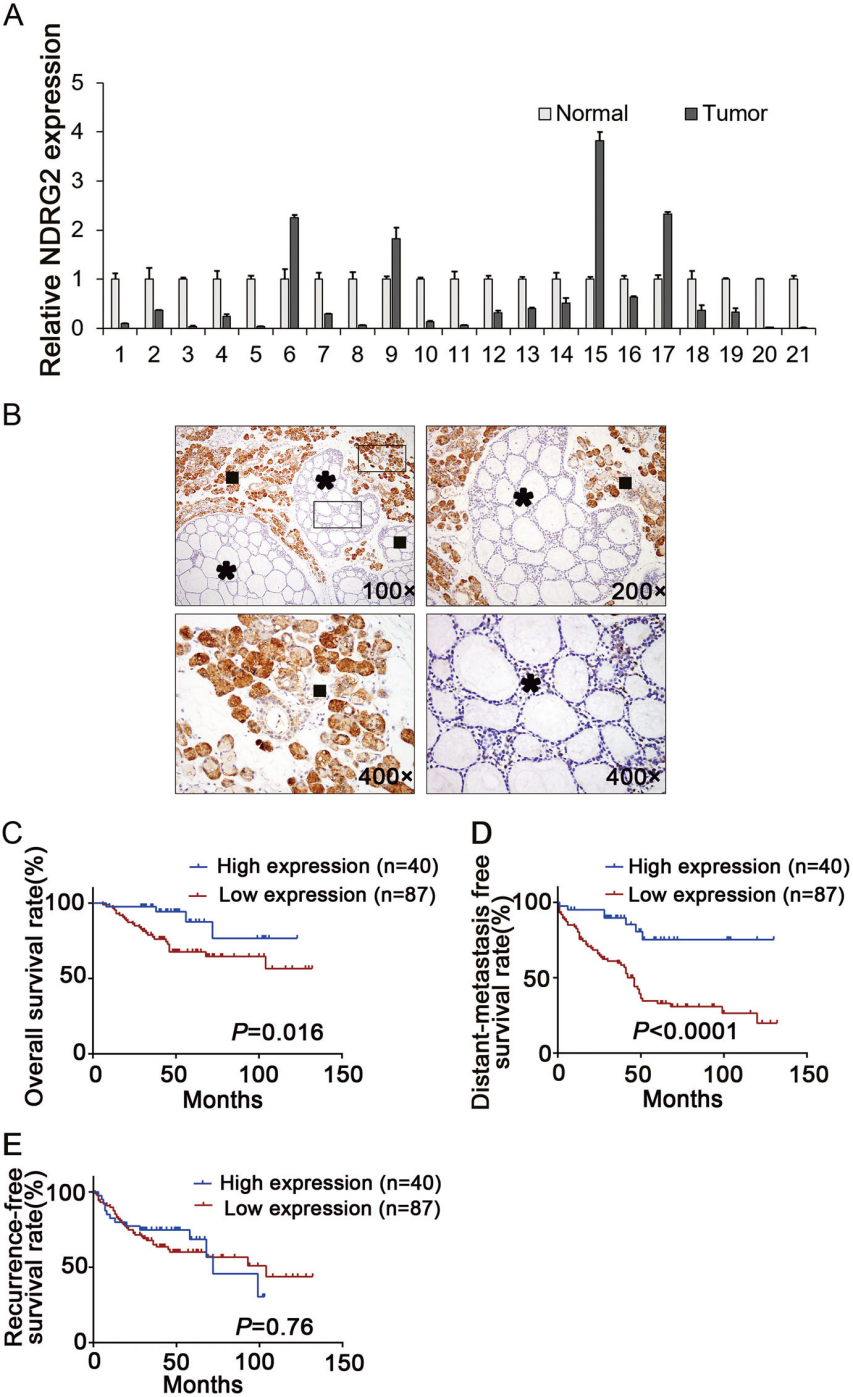
A low NDRG2 expression level is associated with distant metastasis and poorer survival. **a** The relative mRNA expression of NDRG2 in 21 pairs of fresh primary tumor samples and the corresponding normal salivary gland tissues. **b** Representative images of the IHC staining of NDRG2 in paraffin-embedded tissues of tumors and the adjacent normal salivary glands (* tumors, █ normal salivary glands). The Kaplan–Meier analysis was used to compare the overall survival (**c**), distant metastasis-free survival (**d**) and recurrence-free survival (**e**) between the NDRG2-high and NDRG2-negative/low groups of 127 SACC patients

To determine the expression level of NDRG2 in SACC, we tested the relative mRNA expression of NDRG2 by qRT-PCR in 21 fresh primary SACC human tissues and the corresponding normal salivary glands (NSG). A reduction in NDRG2 expression of approximately 1.57- to 109-fold NDRG2 was found in 16 of the 21 (76.2%) SACC tumors, compared with the expression in the surrounding NSG (Fig. 1a).

Then we investigated the protein level of NDRG2 in 127 paraffin-embedded SACC tissues by IHC and analyzed the clinicopathologic parameters. Of the 85 samples that had a matched NSG, 77 (90.6%) exhibited a lower or negative immunostaining for NDRG2 in the SACC tissue than in the corresponding NSG (Fig. 1b). According to the IHC analysis results, the 127 SACC patients were divided into two groups: the NDRG2-negative/low group and the NDRG2-high group. Kaplan–Meier analyses showed that patients with negative/low levels of NDRG2 were inclined to have poorer overall survival (*P* = 0.016) and distant-metastasis-free survival (*P* < 0.0001) rates than those with high NDRG2 levels, but there was no difference in recurrence-free survival between the patients in the two groups (*P* = 0.76) (Fig. 1c–e). Moreover, a statistically significant association between low NDRG2 expression and a high distant metastasis rate was found when analyzing the association between NDRG2 expression level and SACC clinical features (Supplementary Table 1).

### Inhibition of NDRG2 accelerated the proliferation and invasion of SACC cells in vitro

**Fig. 2:**
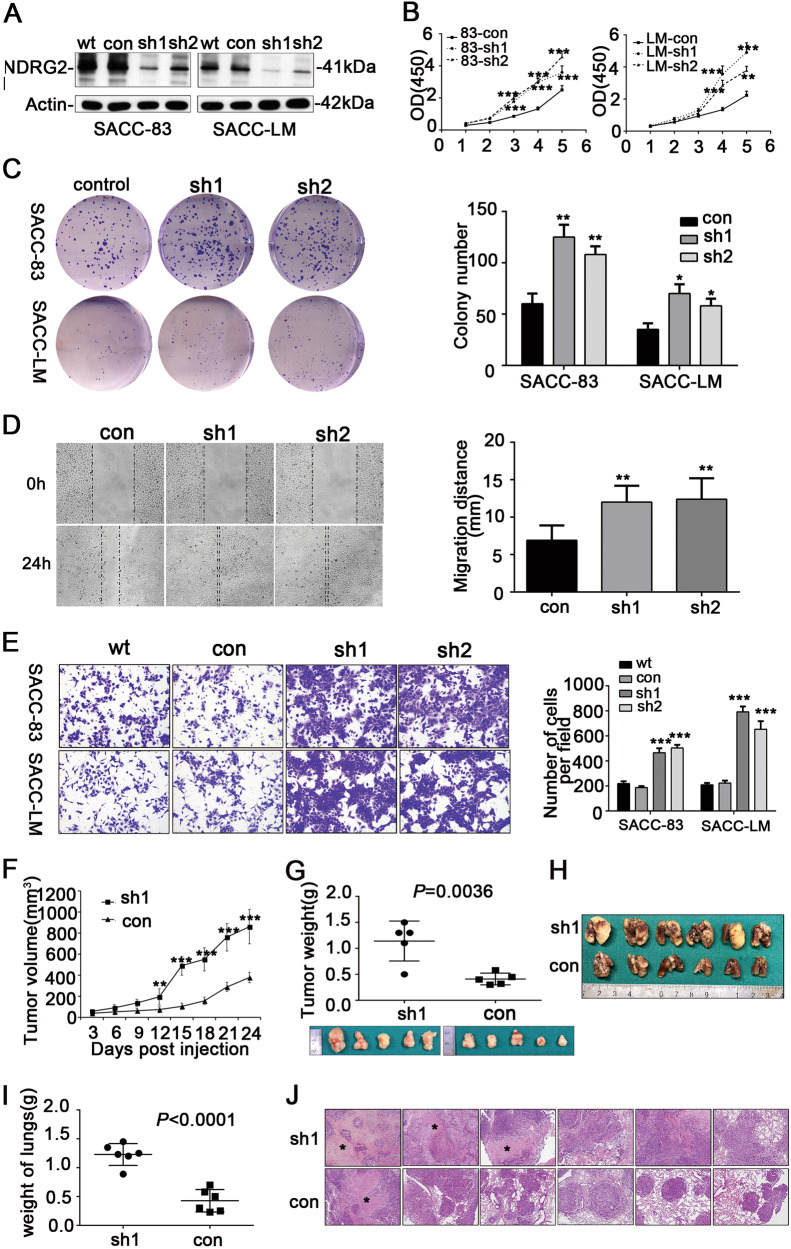
Silencing of NDRG2 promoted cell proliferation, migration and invasion in vitro and in vivo. **a** Attenuated NDRG2 protein expression by knockdown of NDRG2 mRNA. Silencing NDRG2 expression promoted cell growth (**b**) colony formation (**c**) and cell migration as determined by a wound-healing assay (**d**) in SACC cell lines (magnification × 400). Suppression of NDRG2 expression enhanced the invasive capacity (**e**) of SACC cells in a Transwell assay (magnification × 100). **f** Tumor volumes were compared between xenograft tumors (*n* = 5) induced by SACC-LM-NDRG2-sh1 and SACC-LM-con cells. **g** Images of the xenograft tumors arisen from the subcutaneous injection of SACC-LM-NDRG2-sh1 cells and SACC-LM-con cells. Tumor weights were compared between the SACC-LM-NDRG2-sh1 and SACC-LM-con groups. **h** The macroscopic characteristics of metastatic nodules to lungs induced by SACC-LM-NDRG2-sh1 and SACC-LM-con in nude mice (*n* = 6). **i** The weights of lungs with metastatic nodules arisen from SACC-LM-NDRG2-sh1 and SACC-LM-con cells (*P* < 0.01). **j** The histopathologic analysis, by HE staining, of metastases induced by SACC-LM-NDRG2-sh1 and SACC-LM-con cells in lung tissues (original magnifications × 100). (* necrosis). *P < 0.05, **P < 0.01, and ***P < 0.001

To further determine the role of NDRG2 in the biological presentation of SACC, stable clones of SACC-83-NDRG2-sh1/-sh2 and SACC-LM-NDRG2-sh1/sh2 which expressed NDRG2 shRNA and exhibited reduced NDRG2 protein expression were generated (Fig. 2a). The cell proliferation rate was assessed by a CCK8 assay on days 1, 2, 3, 4 and 5; cell growth was strikingly promoted in NDRG2-shRNA-transfected cells compared to the control cells (Fig. 2b). We then preformed colony-formation assay to investigate the tumorigenic potential, and cells expressing SACC-83-NDRG2-sh and SACC-LM-NDRG2-sh displayed a greater number of colonies and larger colonies. Statistically, attenuated NDRG2 expression resulted in an increase in colony number of 1.8–2.1 times in cells expressing SACC-83-NDRG2-sh and 1.6–2.2 times in cells expressing SACC-LM-NDRG2-sh (*P* < 0.01, Fig. 2c).

Our clinical correlation analysis revealed the association of low/negative NDRG2 expression with distant metastasis, so we considered that NDRG2 might involve in SACC cell motility and invasiveness. A wound-healing assay showed that downregulation of NDRG2 enhanced SACC-LM cell migration at the edge of exposed regions (Fig. 2d). Furthermore, Transwell assay demonstrated that the diminished NDRG2 expression in SACC-83-NDRG2-sh and SACC-LM-NDRG2-sh cells led to a dramatical increase in SACC cell invasion. Statistically, inhibition of NDRG2 increased SACC-83 cell invasion by 2.1–2.7 times and SACC-LM cell invasion by 2.9–3.4 times compared with control cell invasion (*P* < 0.01, Fig. 2e).

### Inhibiting NDRG2 promoted xenograft tumor growth and metastatic potential in mouse models

To verify whether NDRG2 knockdown could enhance the tumorigenic potential in vivo, a xenograft tumor mouse model was established by subcutaneously injecting SACC-LM-NDRG2-sh1 and SACC-LM-con cells into the right axillary fossa of nude mice. Throughout the course of 4 weeks, tumor volume was monitored with a caliper, and the group with NDRG2 silencing displayed significantly larger tumors than did the control mice. When the experiment ended, the xenograft tumors were isolated, and the weights were measured; this assay again showed an increased tumor size and weight in mice with NDRG2 downregulation compared with control mice (Fig. 2f, g).

To assess whether repressing NDRG2 could promote cell metastasis in vivo, SACC-LM cells transfected with NDRG2 knockdown or control vector were injected into the tail vein of nude mice. 6 weeks post tail vein injection, the bilateral lungs were removed, weighed and inspected for tumor formation. The size of the eventual metastatic nodes and the weight of lungs with metastases were dramatically affected. Mice injected with SACC-LM-NDRG2-sh1 cells developed massive and confluent metastatic nodules, whereas mice injected with SACC-LM-con cells generated fewer and scattered nodules (Fig. 2h). Statistically, the weights of the lungs from mice injected with SACC-LM-NDRG2-sh1 cells increased approximately 2.2-times compared with those from mice injected with cells expressing the control vector (Fig. 2i). In addition, HE staining of the lungs revealed that the tumor nodules originated from the NDRG2 knockdown cells presented serious tissue destruction and/or necrosis, whereas those derived from the control cells exhibited scattered tumor nodules (Fig. 2j).

### MiR-130a suppressed NDRG2 expression by targeting the 3′UTR of NDRG2

**Fig. 3:**
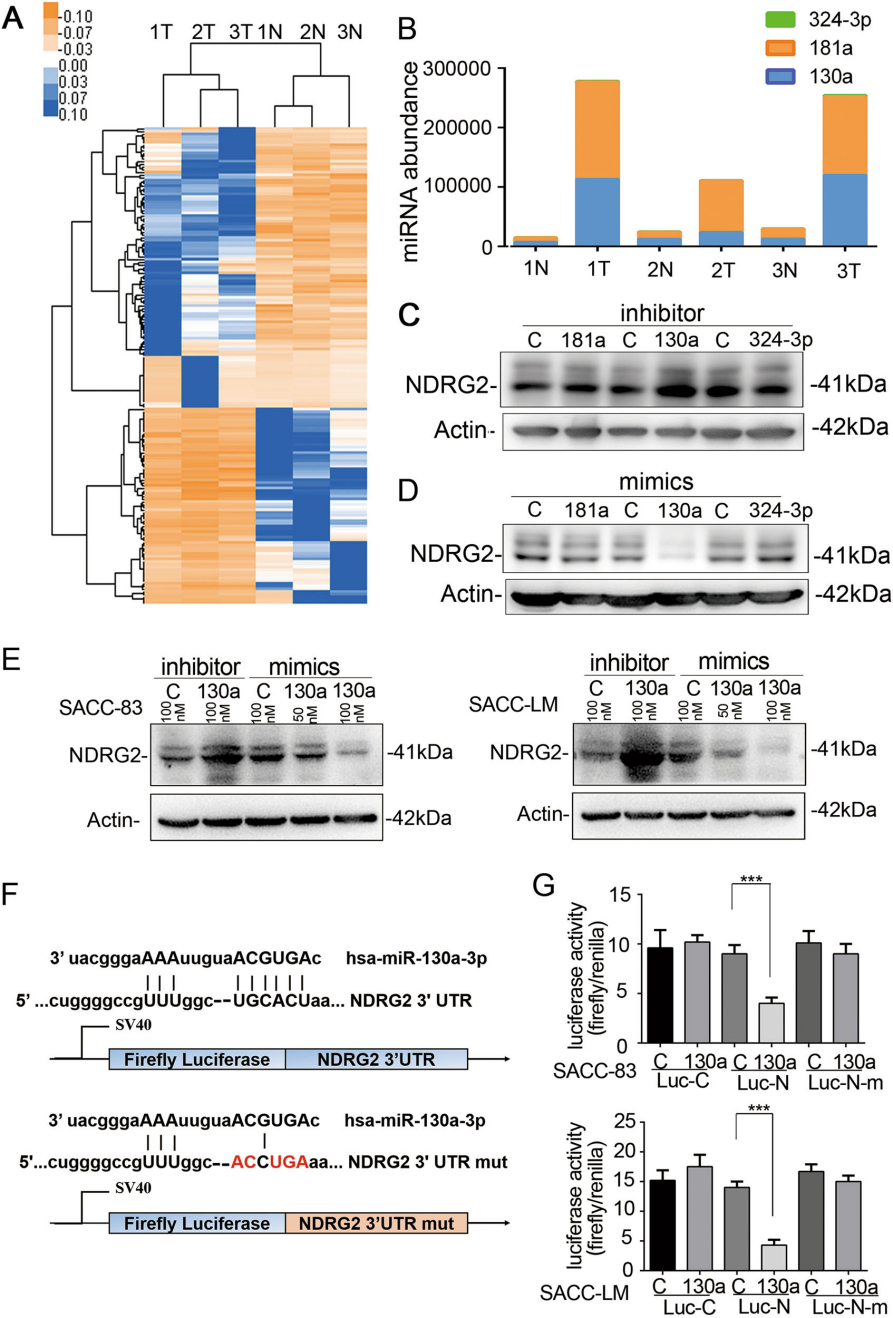
Identification of miRNAs transcriptionally regulating the expression of NDRG2. **a** Hierarchical clustering of gene expression values of the 176 dysregulated miRNAs in the tumor and normal salivary gland samples (log2FC > 1.5 or <−1.5, FDR < 0.05) were charted exhibiting the miRNA expression levels. **b** The abundance of the 3 candidate miRNAs potentially regulating NDRG2 mRNA. NDRG2 was detected by micronome profiling. **c** NDRG2 protein levels were assessed after transfecting the miR-130a inhibitor, miR-181a inhibitor, miR-324-3p inhibitor or inhibitor control into SACC-LM cells. **d** NDRG2 protein levels were assessed after transfecting the miR-130a mimics, miR-181a mimics, miR-324-3p mimics or mimic control into SACC-LM cells. **e** MiR-130a downregulated NDRG2 in SACC-83 (left) and SACC-LM (right) cells in a dose-dependent manner. **f** The wide-type NDRG2 3′UTR sequence and the mutant NDRG2 3′UTR sequence were inserted into the luciferase reporter vector. **g** Forced expression of miR-130a significantly diminished the luciferase reporter activity of the pSiCheck™-2-NDRG2-3′UTR (*P* < 0.01), but not pSiCheck™-2-NDRG2-mutant 3′UTR compared with the miR-con

Base on the cBioPortal for Cancer Genomics (http://www.cbioportal.org/), NDRG2 gene mutation appears to be uncommon in SACC samples and miRNA-mediated NDRG2 regulation in SACC progression remains unknown. To screen miRNAs for potential NDRG2 regulation, we performed micronome profiling in three pairs of SACC samples and the corresponding NSG. After applying a stringent filtering criterion to compare the results from SACC tumor tissue and the adjacent NSG (log2 fold change > 1, FDR < 0.05), we identified 176 dysregulated miRNAs (Fig. 3a). Among the dysregulated miRNAs, 3 candidate upregulated miRNAs, namely miR-130a, miR-181a and miR-324-3p, were predicted to have conserved seed-matching sequences in the NDRG2 3′UTR using RNAhybrid and TargetScan 7.2 analysis (Supplementary Fig. 1). The miRNA abundance in the three pairs of tissues was shown in Fig. 3b. To identify the putative miRNAs targeting NDRG2, inhibitors or mimics of the three miRNAs were transfected into the SACC-LM cell line, and the protein amounts of NDRG2 were analyzed by Western blotting. The results showed that only miR-130a inhibition caused a remarkable increase in NDRG2 protein levels whereas mimics resulted in diminished NDRG2 expression among the three miRNAs in SACC-LM cells (Fig. 3c, d). Furthermore, transfecting both SACC-83 and SACC-LM cells with miR-130a mimics significantly decreased the NDRG2 protein levels in a dose-dependent manner, whereas transfecting the SACC cells with miR-130a inhibitors significantly increased the NDRG2 levels (Fig. 3e).

To make a further validation that miR-130a could directly target NDRG2, dual-luciferase reporter assay was conducted in SACC cell lines using luciferase reporter vectors constructed with the 3′UTR NDRG2 sequence and the mutant NDRG2 3′UTR in which the miR-130a seed-matching sequence was mutated (Fig. 3f). Results showed that forced expression of miR-130a significantly diminished the luciferase reporter activity of the pSiCheck™-2-NDRG2-3′UTR (*P* *<* 0.01), but not pSiCheck™-2-NDRG2-mutant 3′UTR compared with the miR-con (Fig. 3g).

### miR-130a expression was inversely correlated with NDRG2 in SACC samples, and overexpressing miR-130a increased the potential for SACC proliferation and invasion in vitro and in vivo

**Fig. 4:**
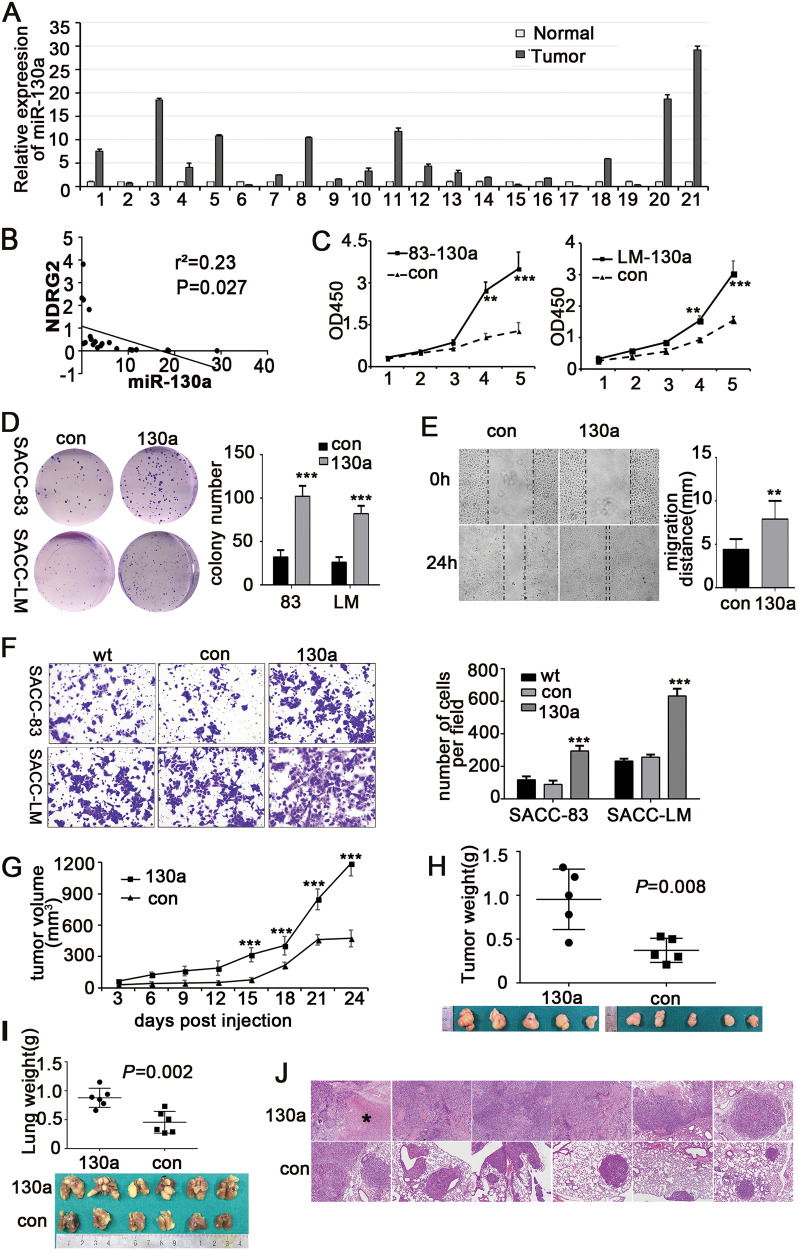
**a** The relative expression of miR-130a in 21 pairs of SACC tumors and the adjacent normal salivary glands. **b** The mRNA relative expression of miR-130a was inversely correlated with NDRG2 in the 21 samples. **c** Effect of miR-130a overexpression on SACC-83 and SACC-LM cell growth. **d** Clonogenic assays showed that miR-130a overexpression significantly increased the colony-forming ability. **e** MiR-130a overexpression enhanced SACC-83 cell migration (magnification ×400). **f** Transwell assay showing that miR-130a overexpression promoted cell invasion in SACC-83 and SACC-LM cells (magnification ×200). **g** Tumor volumes were compared between xenograft tumors (*n* = 5) induced with SACC-LM-130a-con and SACC-LM-miR-con cells. **h** Images of xenograft tumors were shown for the indicated miR-130a-overexpressing and miR-con cells. The weight of the tumors was analyzed. **i** Lung metastasis experiments were performed in mice using lenti-miR-130a cells or miR-con cells. The tumor volume and the bilateral lung weight were analyzed. **j** The histopathologic analysis, by HE staining, of metastases induced by SACC-LM-con and SACC-LM-130a cells in lung tissues (original magnifications ×100). (* necrosis)

MiR-130a can serve as an oncomiR or as a tumor suppressor in distinct cancers. To testify the functional effects of miR-130a on SACC, the expression of miR-130a were analyzed in the 21 primary SACC tumors and corresponding NSG by RT-PCR. An increase in miR-130a expression of approximately 1.58 times to 29.1 times was found in 76.2% (16/21) of the SACC tissues compared with the NSG (Fig. 4a). Furthermore, the negative correlation between the expression of miR-130a and NDRG2 was shown in the 21 SACC tissue samples (*P* = 0.027, Fig. 4b).

Then, we stably overexpressed miR-130a in the SACC cell lines using lentiviral vectors. The transcript level of miR-130a was markedly enhanced, whereas the mRNA and protein level of NDRG2 were significantly diminished in miR-130a-transfected cells compared with those in control cells, which were consistent with the results for the miR-130a mimics (Supplementary Figs. 2–4). CCK8 assay revealed that the aberrant expression of miR-130a promoted proliferation in the SACC cell lines (*P* < 0.05, Fig. 4c). Moreover, the miR-130a-overexpressing cells displayed much greater colony formation ability (an increase of 3.2 times in SACC-83-miR-130a cells and 2.9 times in SACC-LM-miR-130a cells) and enhanced wound healing ability compared with the control cells (Fig. 4d, e). Transwell assays also validated that exogenous miR-130a markedly increased the invasive capacity of both the SACC cell lines (Fig. 4f).

Next, we verified whether miR-130a could accelerate proliferation and metastasis in vivo. Consistent with the results of the in vitro assays, ectopic miR-130a expression enhanced the volume and weight of xenograft tumors formed by the indicated SACC-LM-130a cells (Fig. 4g, h). Moreover, the macro- and microscopic changes in the mouse lungs demonstrated that the metastatic capacity to lungs was significantly enhanced in mice implanted with lenti-miR-130a cells compared to the control mice. The lung weight and number of nodules were drastically increased in mice implanted with miR-130a-overexpressing cells (Fig. 4I, j).

### Restoring the expression of NDRG2 reversed miR-130a-modulated cell proliferation, colony formation and invasion

**Fig. 5:**
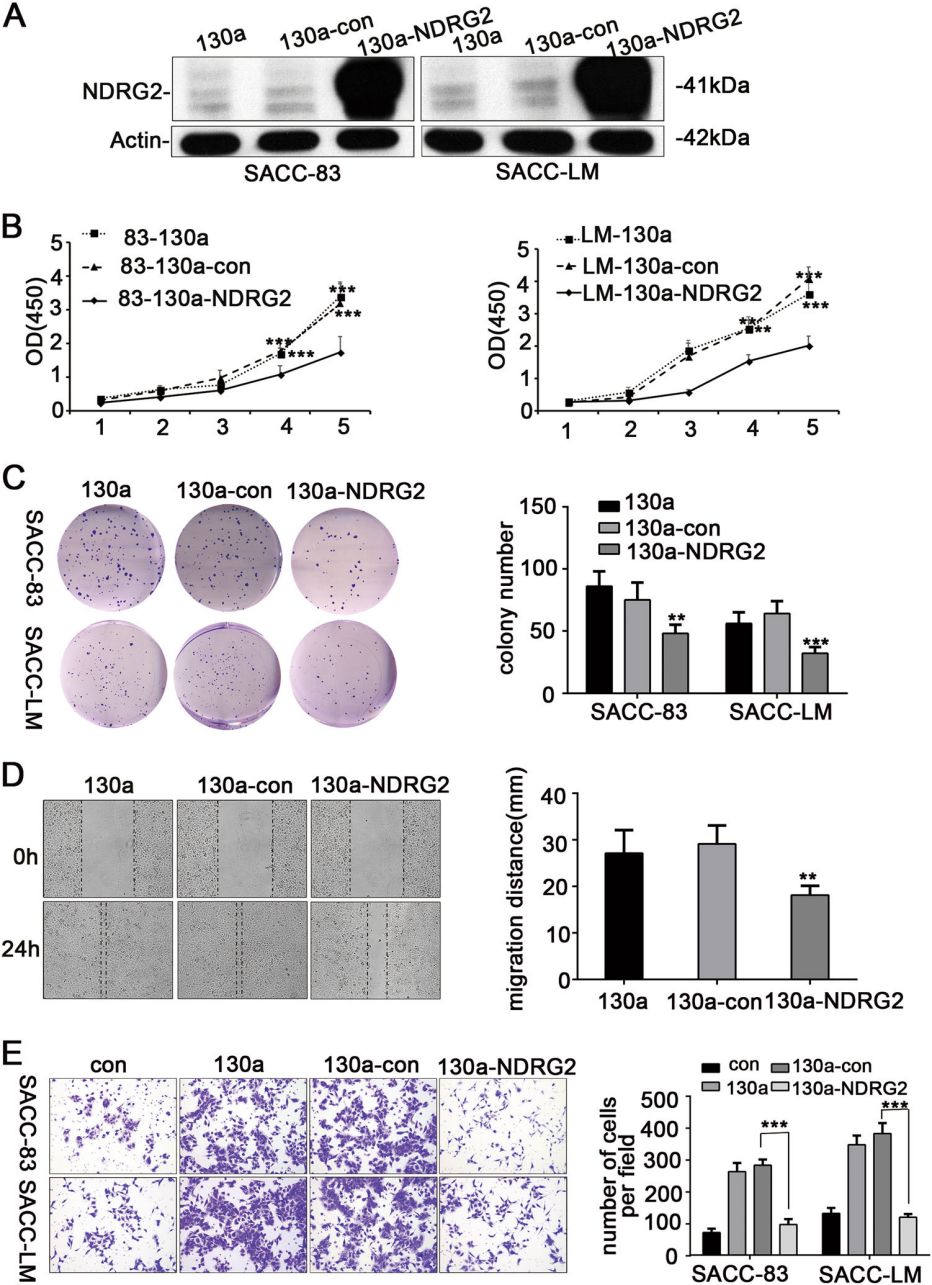
Re-expression NDRG2 reversed the miR-130a-imposed effects on SACC cell proliferation and metastasis in vitro. **a** NDRG2 protein expression was increased by the overexpression of NDRG2 in miR-130a-overexpressing cells. **b** A CCK8 assay showed that NDRG2 overexpression significantly decreased the proliferative ability of miR-130a-transfected SACC cells. **c** Clonogenic assays showed that NDRG2 significantly limited the colony-forming ability of miR-130a- overexpressing cells relative to the that of the negative control. **d** Wound-healing assay indicated that NDRG2 significantly decreased cell motility of miR-130a-overexpresing cells. **e** Transwell assays revealed that NDRG2 significantly suppressed the invasion of miR-130a-transfected cells

To further verify the downregulation of NDRG2 was mediated by miR-130a, a rescue experiment was conducted by stably transfecting a lentiviral expression vector encoding the NDRG2 cDNA sequence into SACC-83-miR-130a and SACC-LM-miR-130a cells. The generated stable clones of SACC-83-130a-NDRG2 and SACC-LM-130a-NDRG2 exhibited upregulated NDRG2 expression compared with control cells (Fig. 5a). Ectopic NDRG2 expression attenuated the promotion in cell growth, colony formation and the invasion resulted from by miR-130a overexpression in the SACC-83 and SACC-LM cell lines. Statistically, forced NDRG2 expression resulted in a decrease in colony number of approximately 1.7-fold in SACC-83-miR-130a cells and of approximately 2.3-fold in SACC-LM-miR-130a cells (Fig. 5b, c); furthermore, re-expression of NDRG2 resulted in a decrease in in vitro cell motility and invasion of approximately 3.6-fold in SACC-83-miR-130a cells and of approximately 2.9-fold in SACC-LM-miR-130a cells (Fig. 5d, e). These data demonstrated that miR-130a could functionally target NDRG2 in SACC and that the loss of NDRG2 in SACC may be attributed to the overexpressed miR-130a.

### The SACC driver gene MYB upregulated miR-130a expression directly and resulted in downregulated NDRG2 expression in human SACC tissues

**Fig. 6:**
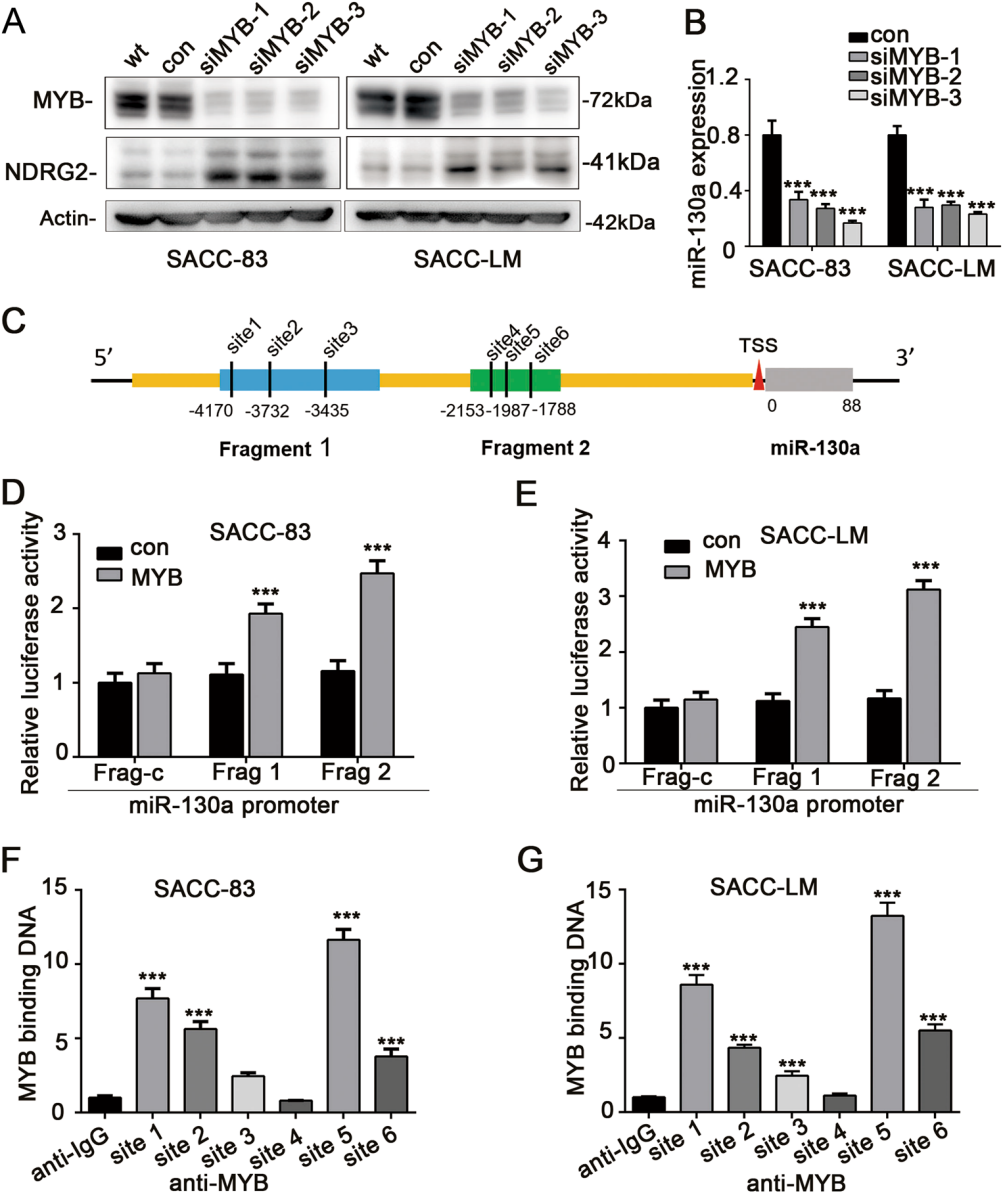
MYB upregulated miR-130a expression directly and induces low protein levels of NDRG2 **a** The protein level of MYB in SACC cells was greatly reduced after transfecting either siMYB. The protein amounts of NDRG2 were significantly increased when MYB was silenced. **b** MiR-130a expression was downregulated in SACC cells when MYB was silenced. **c** Genomic structure of miR-130a: the promoter region was depicted in orange and the pri-miR-130a encoding region was shown in gray. The six potential binding sites were in black. A luciferase reporter assay was conducted in SACC-83 cells (**d**) and SACC-LM (**e**) cells transfected with the PGL3 plasmid constructs with promoter fragment 1 (containing site 1, site 2 and site 3) or promoter fragment 2 (containing site 4, site 5 and site 6) in the presence or absence of pCDNA3.1-MYB. The ChIP qPCR assay displayed that the recruited amounts of MYB binding to either site 1, site 2, site 3, site 5, or site 6 in the miR-130a promoter region were significantly higher than that of negative control IgG in SACC-83 **f** and SACC-LM **g** cells

Previous researches have reported that miR-130a can been transactivated by a subset of transcription factors such as NF-KB and SOX9. Here, we were interested to determine whether miR-130a could be regulated by MYB, the transcription factor that has been widely recognized as the master oncogenic driver in SACC. We achieved MYB knockdown by the transfection of siMYB or control vector into SACC cell lines. Western blotting results testified that the protein level of MYB were significantly decreased, whereas those of NDRG2 were strikingly increased when MYB was silenced compared with control cells (Fig. 6a). Furthermore, miR-130a expression was significantly downregulated by MYB knockdown in both the SACC cell lines (Fig. 6b).

We then verified whether the expression of miR-130a could be directly regulated by MYB. To identify if there were any MYB consensus element in the potential miR-130a promoter region (usually within 5 kb upstream the transcription start site (TSS)), we performed a search of the miRStart and JASPAR databases. There were six putative MYB binding sites in the promoter region as indicated in Fig. 6c. We then assessed the function of the potential transcription binding sites in the miR-130a upregulation by constructing promoter fragment 1 (containing site 1, site 2 and site 3) and promoter fragment 2 (containing site 4, site 5 and site 6) and subjecting them to a luciferase reporter assay. As displayed in Fig. 6d, e, fragments 1 and 2 both yielded robust transcriptional activity induced by MYB. Furthermore, results from the ChIP qPCR assay displayed that the recruited amounts of MYB binding to site 1, site 2, site 3, site 5, or site 6 in the promoter region were significantly higher than that of the negative control IgG, indicating that MYB activated miR-130a expression through binding to the miR-130a promoter at the five potential sites (Fig. 6f, g).

**Fig. 7:**
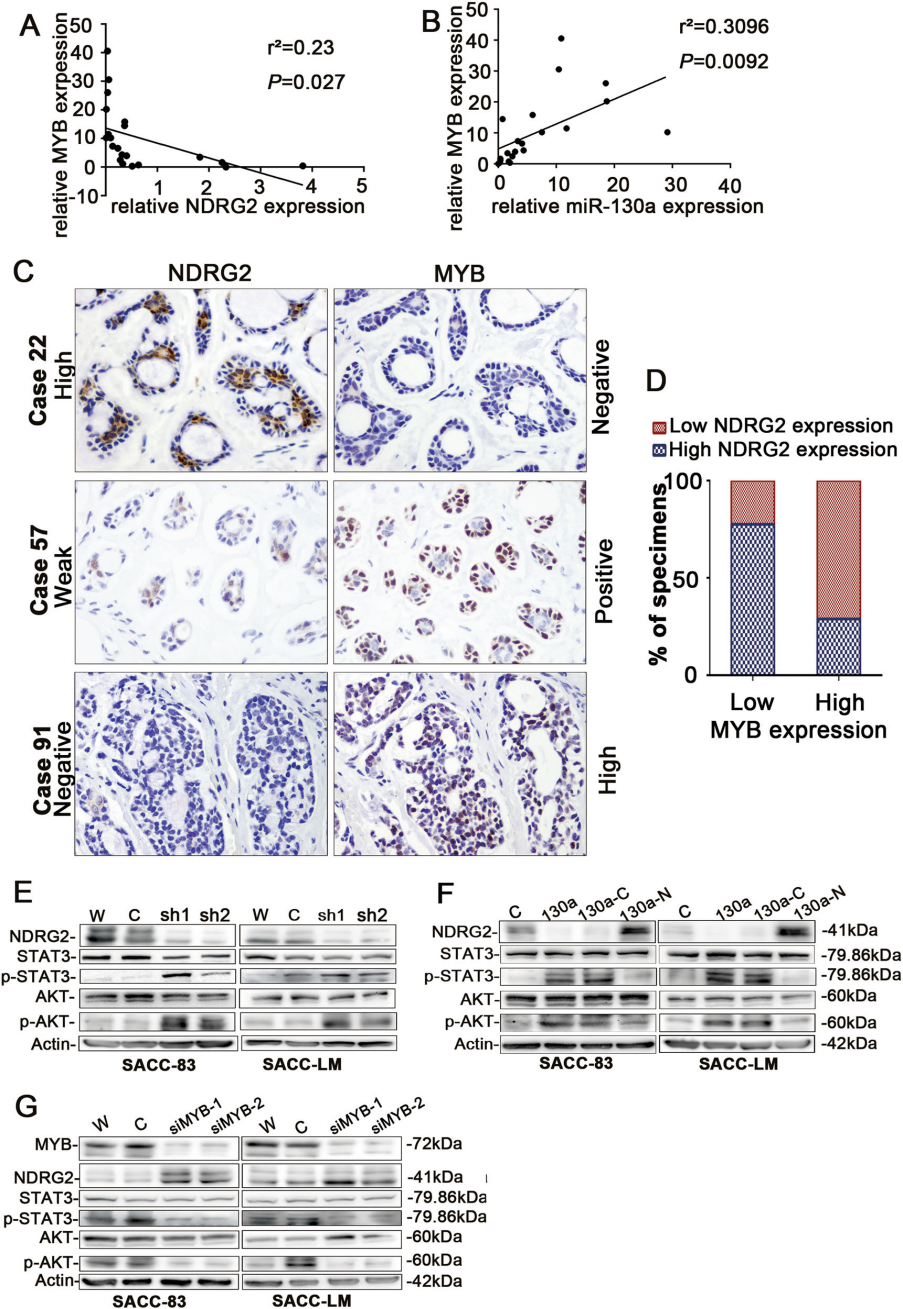
The up-regulated MYB contributed to low expression of NDRG2 in SACC tissues and MYB/miR-130a activates the STAT3 and AKT signaling by targeting NDRG2. **a** The correlation between MYB and NDRG2 in fresh SACC specimens was analyzed. **b** The correlation between MYB and miR-130a in fresh SACC specimens was analyzed. **c** The expression of NDRG2 was negatively correlated with MYB in clinical SACC specimens. Three representative cases were shown. Strong nuclear MYB immunoreactivity was often restricted to tumor myoepithelial tumor cells and weak cytoplasm NDRG2 immunoreactivity was typically observed in luminal tumor cells in tubular foci. (magnification ×200). **d** The histogram displayed percentage of samples exhibiting low or high MYB expression in relation to NDRG2 expression levels. **e** The pSTAT3, pAKT, STAT3, and AKT expression levels in SACC-83 and SACC-LM cells transfected with sh-NDRG2 or sh-con plasmids were examined. Both pSTAT3 and pAKT were upregulated when NDRG2 was silenced. **f** The restoration of NDRG2 expression reversed the miR-130a-induced pSTAT3 and pAKT upregulation in the SACC-83 and SACC-LM cell lines. **g** MYB knockdown activated NDRG2 and decreased the expression of pSTAT3 and pAKT

In order to further validate the effect of MYB/miR-130a/NDRG2 axis in SACC, the mRNA expression was first examined in the 21 pairs of fresh SACC samples and NSG using RT-PCR and determined the association among miR-130a, NDRG2 and MYB (Supplementary Fig. 5). The results showed that the relative expression of MYB had a positive correlation with miR-130a expression, whereas NDRG2 expression had an inverse correlation with MYB expression (Fig. 7a, b). Then we explored whether there might be a potential correlation between protein levels of NDRG2 and MYB in paraffin-embedded SACC tissue samples. Overall, 70.11% (61 cases) of specimens with high MYB expression (87 cases) exhibited a low/negative level NDRG2, whereas 77.5% (31 cases) of specimens with low MYB expression (40 cases) showed high expression of NDRG2 (Fig. 7c, d). Most interestingly, in SACC with tubular growth pattern, the inner luminal epithelial cells presented a negative staining for MYB but a weak positive staining for NDRG2, whereas the outer myoepithelial cells exhibited positive staining for MYB but a negative staining for NDRG2 (Fig. 7c, Case 57). These data indicated that in SACC, upregulated MYB induced low expression of NDRG2 and overexpressing NDRG2, thereby inducing resistance to the oncogenic effects of MYB and miR-130a, presents a promising strategy for treating SACC.

### MYB/MiR-130a activated the STAT3 and AKT signaling by targeting NDRG2

To figure out the potential mechanisms underlying the tumor promotion effects of MYB/miR-130a, downstream signaling of NDRG2 was investigated. As shown in Fig. 7e, f, NDRG2 knockdown or forced miR-130a expression led to an increase in the protein amounts of phosphor-tyr705 of STAT3 (pSTAT3) and phosphor-AKT (pAKT) but not total STAT3 or AKT. However, restoration of NDRG2 in exogenous miR-130a expression cells contributed to a reduction of pSTAT3 and pAKT protein level compared with SACC-miR-130a-con cells (Fig. 7f). Moreover, MYB downregulation inducing higher NDRG2 expression decreased the expression of pSTAT3 and pAKT but not total STAT3 or AKT (Fig. 7g). Overall, the results suggested that MYB/miR-130a activated the STAT3 and AKT signaling pathways by targeting NDRG2.

## Discussion

SACC is a relentless and aggressive malignancy characterized by high rates of relapse, a tendency toward perineural invasion and distant metastasis, and poor long-term patient survival^16^. Currently, the treatment options for SACC are restricted to surgery with or without radiation^17^. Resistance to chemotherapy and radiation resulting in the high risk of recurrence and dismal overall survival of SACC emphasizes the demand for molecular target therapies^18^. To date, no single targeted drug has been approved by the Food and Drug Administration (FDA) for SACC^19^. Therefore, new therapeutic targets are significant to improve curative efficacy of SACC. In our research, we found that the MYB/miR-130a/NDRG2 axis played a crucial part in the SACC proliferation and metastasis, indicating the potential to act as therapeutic targets in SACC.

NDRG2, which strongly influences cell-cycle arrest, metabolic reprogramming, senescence and apoptosis, is known as a pleiotropic tumor suppressor in several malignancies^20–22^. However, the biological role of NDRG2 and its underlying molecular mechanisms are not well understood. In this study, we identify the function of NDRG2 in SACC growth and metastasis for the first time. NDRG2 inhibits the proliferation and colony formation ability and decreases the metastatic potential of SACC cells in vitro and in vivo. Most importantly, the results of our study show that low expression of NDRG2 is associated with poorer OS and DFS in SACC patients, suggesting that NDRG2 plays a critical role in maintenance of the proliferative and metastatic characteristics of SACC and has the great potential to be a new molecular target in treating metastatic tumors.

MiRNA dysregulation is frequently observed in cancer, but only a few of them were identified to play prominent roles in the carcinogenesis and progression of SACC. These miRNAs include upregulated miR-21^23^ and miR-17-92^24^, and downregulated miR-125-5p^25^, miR-98^26^, miR-101-3p^10^, and miR-320a^27^. In the present study, a micronome-based differential miRNA profiling was performed to screen the possible miRNAs targeting NDRG2. After a miRNA target prediction algorithm analysis, three upregulated miRNAs, namely miR-130a, miR-181a and miR-324-3p, were identified to potentially target the 3′UTR of NDRG2. We then validated that only miR-130a overexpression apparently induced the reduction of the endogenous NDRG2 levels and inhibition of miR-130a significantly upregulated NDRG2 in SACC cells; furthermore, we demonstrated the complementary binding between miR-130a and the NDRG2 3′UTR using a luciferase reporter assay.

MiR-130a exerts different biological effects in different malignancies, depending on its target mRNAs^28,29^. One previous study based on SACC cell lines that focused on microRNA profiling and target genes related to the metastasis observed that SACC cells showed a higher gene expression of miR-130a^30^. However, further evidence should be provided to fully elucidate the biological role and mechanism of miR-130a in SACC aggressiveness. In our study, we found that miR-130a was upregulated in clinical human SACC samples; interestingly, NDRG2 was inversely correlated with miR-130a expression. Moreover, the forced miR-130a expression strikingly increased SACC cell proliferation and invasion in vitro, and dramatically enhanced their ability to promote tumor growth and seed lung metastases in vivo. In addition, restoration of NDRG2 rescued the miR-130a-imposed impetus for tumor proliferation and metastasis. Taken together, our research not only validated the biological function of miR-130a in promoting SACC proliferation and metastasis but also demonstrated the protumor mechanism of miR-130a by directly targeting NDRG2.

Recently, it has been shown that the interactions between transcription factors and miR-130a are critical in many pathologic conditions. In cervical carcinoma cells and biliary epithelial cells, NF-KB was found to upregulate miR-130a transcriptionally^31,32^. In addition, SOX9 could inversely regulate miR-130a to affect cervical cancer chemoresistance^33^. The transcription factor MYB has been widely accepted to act as a critical oncogenic driver of SACC^34^. However, the molecular mechanism of how MYB underlies tumor progression remains unclear. Our results uncovered the mechanism of MYB in driving SACC aggressiveness through the positive regulation of miR-130a and the decrease in the downstream NDRG2 expression levels. Most importantly, through modulating the STAT3 and AKT pathways, NDRG2 can play an anti-cancer role downstream MYB/miR-130a in SACC.

In the cohort of 21 SACC patients, an exceptional case of patient 9 is noticed with high expression of NDRG2, miR-130a and MYB, which at the same time does not seem to match the hypothesis like most of the other patients. The reasons underlying are really complex. One possible reason might be that SACC displays inter-tumor heterogeneity and no module or axis is found to be functional for all SACC patients. For patient 9, other effective factors might involve in the expression of NDRG2 during the cancer progression. In addition, miR-130a mimics were demonstrated to significantly decrease NDRG2 protein levels in a dose-dependent manner in our study. We found that in the cohort of the 21 patients, 16 tumors showed an increase in miR-130a expression compared with the NSG. However, patient 9 exhibited the lowest ratio (1.58 times) among the 16 cases. The miR-130a expression level in this patient may be not high enough to suppress NDRG2.

In conclusion, our results provide the first evidence that NDRG2 is an important antioncogene that contributes to the inhibition of SACC growth and metastasis. Downregulation of NDRG2 is the consequence of miR-130a overexpression in SACC. In addition, miR-130a upregulation is attributed to MYB overexpression. There is a MYB/miR-130a/NDRG2 axis that results in SACC progression. Our findings have enriched the body of knowledge about the molecular mechanisms underlying SACC and provide potential targets for future therapeutic invention.

## Methods

### Cell culture

The SACC-83 cell line was derived from sublingual gland of a SACC patient; SACC-LM cells with higher metastatic abilities to lungs were isolated after injecting SACC-83 cells into the tail vein of nude mice^35,36^. The cell lines were subjected to DNA STR profiling by Biowing Applied Biotechnology Co., Ltd. (Shanghai, China) to avoid misidentification and cross-contamination. HEK-293T cells were achieved from the China Type Culture Collection (Shanghai, China). All cell lines were cultured in DMEM which contained 10% FBS at 37 °C with 5% CO_2_.

### Clinical specimen collection

All the SACC samples were gathered from patients who experienced surgery at the Ninth People’s Hospital affiliated to Shanghai Jiao Tong University. Twenty-one fresh primary SACC tissue samples and the surrounding normal salivary gland tissue were obtained between 2015 and 2016 and were subjected to qRT-PCR analysis. A total of 127 paraffin-embedded SACC specimens for which complete follow-up records were available for the corresponding patients were obtained between 2000 and 2014 and investigated with IHC; 85 of these samples included matched tissues from the surrounding normal salivary gland or normal submandibular gland obtained during neck lymph node dissection. Ethical approval was acquired from Independent Ethics Committee of Shanghai Ninth People’s Hospital affiliated to Shanghai Jiaotong University, and each patient signed the informed consent.

### Plasmids and lentivirus transfection

To knockdown NDRG2, the pGMLV-SC5 RNAi lentiviral vector was purchased from Shanghai Genomeditech. By inserting the specific shRNA sequences into the PGMLV-SC5 vector, the pGMLV-NDRG2-sh1 and pGMLV-NDRG2-sh2 plasmids were generated. The shRNA sequence pairs were as follows: NDRG2-sh1, gatccGAGATGATCCTTGGACATCTTCTCGAGAAGATGTCCAAGGATCATCTC

TTTTTT and aattAAAAAAGAGATGATCCTTGGACATCTTCTCGAGAAGATGT

CCAAGGATCATCTCg; NDRG2-sh2, gatccGCCTACATCCTGGCGAGATATCTCG

AGATATCTCGCCAGGATGTAGGCTTTTTT and aattAAAAAAGCCTACATCCTGGCGAGATATCTCGAGATATCTCGCCAGGATGTAGGCg; sh-con, gatctGTTCTCCGAACGTGTCACGTTTCAAGAGAACGTGACACGTTCGGAGAATTTTTTc and aattgAAAAAATTCTCCGAACGTGTCACGTTCTCTTGAAACGTGACACGTTCGGAGAACa.

For the miR-130a and NDRG2 overexpression study, pri-miR-130a sequences were synthesized by Shanghai Sangon Biotech, and the NDRG2 gene was PCR-amplified according to the NDRG2 cDNA sequences. The lentiviral expression vector PGMLV-CMV-MCS-EF1-ZsGreen1-T2A-Puro was purchased from Shanghai Genomeditech. The pri-miR-130a and NDRG2 cDNA sequences were inserted into the vector respectively to generate PGMLV-miR-130a and PGMLV-NDRG2 plasmid. Lentivirus packaging and transfection were performed according to Ref. ^37^.

### Transfection of miRNA mimics, inhibitors, and siRNAs

The miRNA mimics, inhibitors and siRNAs targeting MYB were designed and purchased from Shanghai Genomeditech and then transfected into Cultured SACC-83 and SACC-LM cells with the Lipofectamine 2000 reagent (Invitrogen.) following the manufacturer’s protocol. The sequences of the miRNA mimics, inhibitors and siRNAs targeting MYB were described in Supplementary Table 3.

### Immunohistochemistry

We performed Immunohistochemistry staining with MYB and NDRG2 antibody using the Envision^TM^ detection kit (Dako, USA) according to the manufacturer’s instructions. According to the ref. ^38^, NDRG2 expression level were considered as high if ≥10% of the cells showed moderate to strong staining, low if either cytoplasmic or nuclear staining was noted in <10%, and negative if neither cytoplasmic nor nuclear staining was seen. The samples were divided into two groups: the first group comprised the samples for which the NDRG2 expression score was high, and the second group comprised the rest of the samples (NDRG2 low/negative group). MYB staining was scored as high if more than 5% of tumor cells exhibited strong nuclear immunoreactivity^39^. Each data set was analyzed separately, and a consensus in the evaluation from at least two of the three investigators were considered acceptable.

### Micronome profiling

Total RNA was extracted from each sample using Trizol reagent and purified by miRNeasy Mini Kit (Qiagen). Then we prepared the small RNA complementary DNA libraries using the TruSeq Small RNA Sample Prep Kit (Illumina, USA). In brief, 1ug small RNA were added to 3′ and 5′ adaptors, reverse transcribed to cDNA and amplified using PCR. 145–160 bp library sequences were then obtained after size selection. All profiling work was conducted with the help of Shanghai NovelBio Bio-Pharm Technology Co., Ltd. On Hiseq2000 platform. After filtering and mapping the clean reads to miRNA database, we applied limma algorithm to achieve the differentially expressed genes after the significance analysis with the following criteria: (1) log2 Fold Change > 1; and (2) FDR < 0.05.

The micronome data were deposited in the Gene expression omnibus (GEO) database. The accession number is GSE117275.

### Cell growth and colony formation assays

Cell counting kit-8 (CCK-8) assay was used to evaluate cell growth by following the manufacturer’s protocols (Dojindo, Tokyo, Japan). Briefly, transfected cells were collected and seeded into 96-well plates at 1 × 10^3^ cells per well in triplicate. Following days 1, 2, 3, 4 and 5 of growth in the previously described growth conditions, 10 µl of CCK8 assay solution was added to each well. After incubation for 2 h at 37 °C, the values of optical density at 450 nm were evaluated.

For the colony formation assay, 1 × 10^3^ cells were seeded into 6-well plates per well in triplicate. Two weeks later, the colonies were fixed and stained with 0.5% crystal violet. Under the microscope, the number of colonies which consist of more than 50 cells was calculated.

### Cell invasion

Cell invasion was quantified using SACC cell lines in Transwell chambers (24-well insert; 8 µm pore size; Corning Incorporated, USA). BD Matrigel™ (BD Biosciences Inc., USA) was thawed at 4 °C and then diluted with cold DMEM at a ratio of 1:5. Twenty-five microliters of the diluted Matrigel matrix was added to the Transwell membrane inserts. When the plates were incubated for 6 h at 37 °C, 2 × 10^5^ cells in 200 µl of serum-free DMEM were seeded onto the Matrigel-coated upper chamber, and the lower chamber was filled with 500 µl of DMEM with 10% serum. After 24 h invading, the cells on the lower side of the chamber were fixed with methanol for 10 min and stained with 0.5% crystal violet for 15 min. Five random fields on the lower side of the chamber were selected for quantification under an inverted microscope (200 × ).

### Xenograft formation and in vivo metastasis assay

The animal experiments were conducted following the ethical standards and national guidelines and were approved by the Committee on Ethics of Animal Experiments of the Ninth People’s Hospital Affiliated to Shanghai Jiaotong University. The animal care and treatment followed the institutional guidelines. Female BALB/c-nu/nu nude mice, aged 5–7 weeks, were purchased from Vital River Laboratory (Beijing, China) and were kept under standard conditions. To establish the xenograft model, SACC-LM cells (2 × 10^6^) resuspended in 100 μL of PBS were injected into the right axillary fossa of each mouse. The tumor size was measured every 3 days for 4 weeks and was calculated using the formula *V* = width^2^ × length/2. When the experiment ended, the tumors were collected and weighed.

For the intravenous mouse model, 2 × 10^6^ cells were suspended in 100 μL of PBS and injected into the tail vein of nude mice. After 6 weeks, the mice were sacrificed, and the tumors nodules formed on the bilateral lungs were counted. The lungs were collected and paraffin-embedded for further hematoxylin and eosin staining.

### RNA isolation and RT-qPCR analysis

Total RNA was isolated from SACC cells and tissues with the mirVana miRNA Isolation Kit (Ambion, Texas, USA). For the NDRG2 RT-qPCR analysis, the first-strand cDNA synthesis kit (TaKaRa Code: RR037A) was used to make a reverse transcription. The Mir-X^™^miRNA First Strand Synthesis Kit was used to convert miRNAs into cDNA (Clontech Code: 638315). The qPCR experiments were carried on an ABI7500 FAST system using TB Green™ Premix Ex Taq™ II (Takara Code: RR820A). The mRNA and miRNA expression levels were respectively normalized to β-actin and U6 expression. The differential expression of miRNA and mRNA was calculated by the 2^-ΔΔCt^ formula, where ΔΔCt = ΔCt for the treatment cells –ΔCt for the control cells. All PCR analyses were performed in triplicate. The primers used were as follows: NDRG2, 5′GAGATATGCTCTTAACCACCCG3′ and 5′GCTGCCCAATCCATCCAA3′; and miR-130a, 5′acgCAGTGCAATGTTAAAAGGGCAT3′.

### Western blot analysis

Cells were lysed with RIPA lysis buffer (Solarbio, Beijing) supplemented with PMSF and a protease inhibitor mixture (Roche Diagnostics, Switzerland). A bicinchoninic acid protein assay was used to measure the protein concentrations (Thermo Fisher Scientific, Inc.). After isolation with 10% SDS-PAGE, the protein was transferred to PVDF membranes (Millipore, USA). When blocked with 5% fat-free milk for 1 h, the membranes were incubated with primary antibodies (Supplementary Table 2) at 4 °C overnight and then with horseradish peroxidase (HRP)-conjugated secondary antibody (PMK biotechnology, Wuhan, China) for 2 h at room temperature. The immunoreactive bands were visualized using ECL Plus reagents (Pierce, Thermo Fisher Scientific, Inc.).

### Luciferase assay

To identify whether miR-130a could regulate NDRG2 directly, NDRG2 3′UTR with wild-type or mutant miR-130a binding sites were amplified by PCR and the fragments were inserted into the pSiCheck™-2 vector (Promega, USA) respectively. The dual luciferase assays were performed in 96-well plates in triplicate. 200 ng of each construct or control vector was cotransfected with the PGMLV-miR-130a plasmid (50 nM) or the PGMLV vector into HEK-293 cells using Lipofectamine 2000.

For investigating whether MYB can regulate the miR-130a pri-miRNA directly, the full-length MYB cDNA sequence was PCR amplified and cloned into the pCDNA3.1 vector to generate pCDNA3.1-MYB. The miR-130a promoter fragment 1 and fragment 2 were synthesized by Sangon Biotech and were inserted into the pGL3-promoter vector. SACC cell lines were transfected with 50 ng of either pGL3-promoter fragment 1, pGL3-promoter fragment 2 or pGL3-promoter in the presence of pCDNA3.1-MYB (50 ng) using Lipofectamine 2000 (Invitrogen). The Dual-Glo™ Luciferase Assay System (Promega) were then used to measure the relative luciferase activity in lysates after 48 h transfection.

### Chip qPCR assay

The ChIP assay was performed using a commercial EZ ChIP^TM^ kit (Millipore, USA) according to the manufacturer’s protocol. An antibody against MYB was used to immunoprecipitate the MYB-chromatin complexes from cell extracts. The immunoprecipitated DNA was purified and tested by qPCR. Sequences of primer pairs are listed in Supplementary Table 3.

### Statistical analysis

The statistical analyses were processed using the Graphpad Prism 6 software or SPSS 17.0 statistical software. Values of three independent experiments were shown as the mean ± SD; paired Student’s *t*-test was used to compare groups. A Chi-squared test was performed to examine the association between NDRG2 expression and SACC clinicopathological parameters. Overall survival, distant metastasis-free survival and recurrence-free survival were compared with the Kaplan–Meier method. To calculate the correlations, the Pearson correlation coefficient was used, and *P*-values < 0.05 were considered statistically significant.

## Electronic supplementary material

SACC-LM cell line authentication

supplementary data

SACC-83 cell line authentication

Supplementary figure legends
