# Supplementary material for: The MYB/miR-130a/NDRG2 axis modulates tumor proliferation and metastatic potential in salivary adenoid cystic carcinoma
Source: Cell Death Dis. 2018 Sep 11;9(9):917. doi: 10.1038/s41419-018-0966-2 (PMC6134089; doi:10.1038/s41419-018-0966-2)
Supplement: Supplementary file 2 — supplementary data [file 41419_2018_966_MOESM2_ESM.pdf]

Supplementary Table 1: Correlation Between NDRG2 Expression and Clinicopathologic Parameters of Adenoid Cystic Carcinoma

Characteristics	NDRG2 expression		*P* value
	high	low	
Age, y			0.794
≤55	22	50	
>55	18	37	
Sex			0.21
Male	15	44	
Female	25	43	
Tumor site			0.261
Major glands	15	24	
Minor glands	25	63	
Histological grade			0.713
I-II	33	74	
III	7	13	
Perineural invasion			0.667
Yes	27	62	
No	13	25	
Recurrence			0.783
Yes	11	26	
No	29	61	
Distant metastasis			.000
Yes	7	63	
No	33	24	

Supplementary Table 2: primary antibodies used in this study

Protein	Assay	Antibody
NDRG2	WB, IHC	sc-376202, Santa Cruz
MYB	WB	Ab-45150, Abcam
Stat3	WB	9139, CST
p-Stat3	WB	52075, CST
AKT	WB	4685s, CST
p-AKT	WB	4051, CST

Supplementary Table 3: Sequences of primers, miRNA mimics, inhibitors and siRNAs

Name	Sequences(5’-3’)
hsa-miR-130a mimic	CAGUGCAAUGUUAAAAGGGCAU
	AUGCCCUUUUAACAUUGCACUG
hsa-miR-181a inhibitor	ACUCACCGACAGCGUUGAAUGUU
hsa-miR-324a-3p inhibitor	CCAGCAGCACCUGGGGCAGUGGG
hsa-miR-130a inhibitor	AUGCCCUUUUAACAUUGCACUG
mimic-control	UCACAACCUCCUAGAAAGAGUAGA
	UCUACUCUUUCUAGGAGGUUGUGA
inhibitor-control	UCUACUCUUUCUAGGAGGUUGUGA
siMYB-1	AAAGUACUAAACCCUGAGC tt
	GCUCAGGGUUUAGUACUUU tt
siMYB-2	CAAUGUUCUCAAAGCAUUU tt
	AAAUGCUUUGAGAACAUUG tt
siMYB-3	CUUGGAAAUGCCUUCUUUA tt
	UAAAGAAGGCAUUUCCAAG tt
siRNA-control	UUCUCCGAACGUGUCACGUdTdT
	ACGUGACACGUUCGGAGAAdTdT
Promoter-site-1-F	CAAGGAAAATGATGTCTTTGGAGC
Promoter-site-1-R	GGAGAGAGAACAACACAAAGGCT
Promoter-site-2-F	AGAAGCAAGACTGGACAAACAC
Promoter-site-2-R	TTGGAGGATAAGGAGGGCAG
Promoter-site-3-F	TGGGCTGTCCATCTTCTTGT
Promoter-site-3-R	GAGCACACACATACATGTACATCAT
Promoter-site-4-F	ATTAAAAAGCCACTTCCTCAGTCAC
Promoter-site-4-R	CCTGATCTTGCAAGACTGTCATTAA
Promoter-site-5-F	CATCTAAACCCAAAGAATGGACC
Promoter-site-5-R	TGACCCCATAGTACTCAGCCTAT
Promoter-site-6-F	GCATTTTGTTGCAGCCCATA
Promoter-site-6-R	TAGCTGGCATCTGCTGGTTG

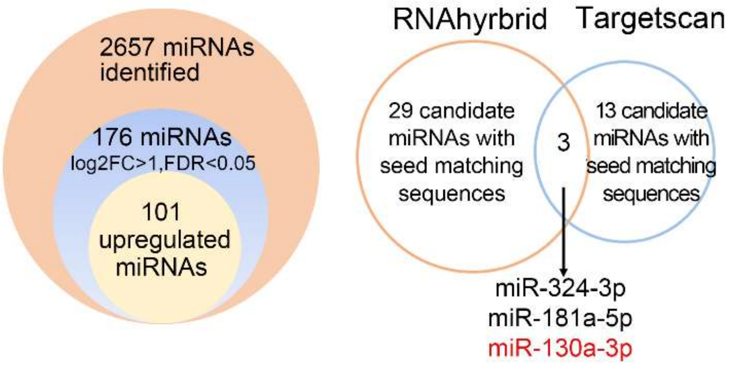

Supplementary Figure 1: Venn diagrams showing the number of total mapped miRNAs in both groups, the number of differentially expressed miRNAs and the three candidate miRNAs regulating NDRG2 in the two groups.

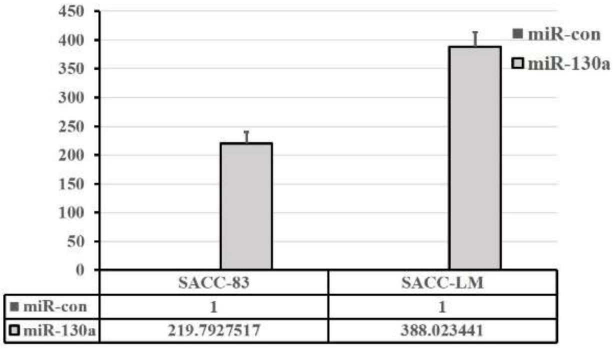

Supplementary Figure 2: The relative miR-130a levels in miR-130a over-expression cells and the control cells.

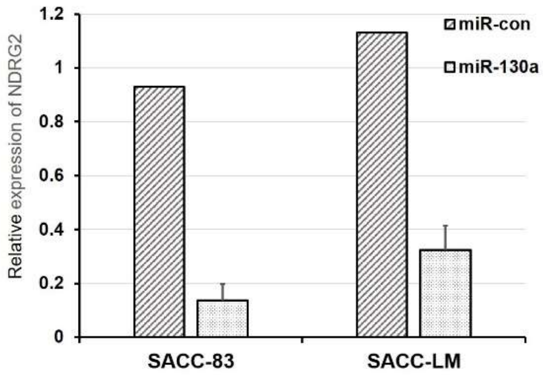

Supplementary Figure 3: The relative mRNA levels of NDRG2 in miR-130a over-expression cells and the control cells.

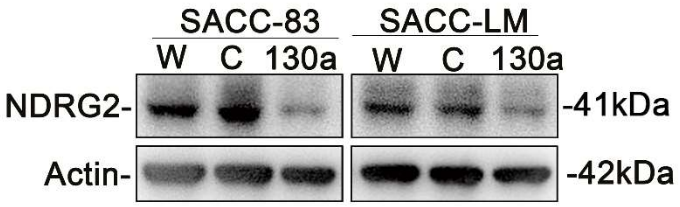

Supplementary Figure 4: the reduction of NDRG2 protein amounts in miR-130a-overexpressing cell lines.

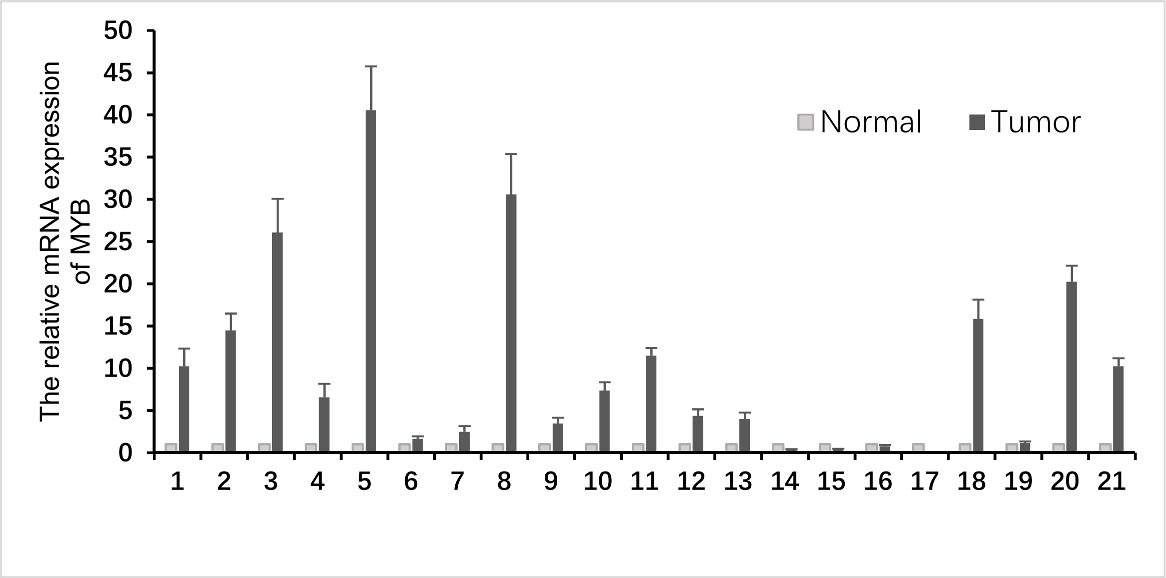

Supplementary Figure 5: the relative expressions of MYB in tumors and the corresponding normal salivary gland of fresh samples.
