# Supplementary material for: The MYB/miR-130a/NDRG2 axis modulates tumor proliferation and metastatic potential in salivary adenoid cystic carcinoma
Source: Cell Death Dis. 2018 Sep 11;9(9):917. doi: 10.1038/s41419-018-0966-2 (PMC6134089; doi:10.1038/s41419-018-0966-2)

Sample File	Sample Name	Panel	OS	SQ
50_B07_XiBaoJianDing0318-1-0320.fsa	XRH83	STR Profile 1-dup		

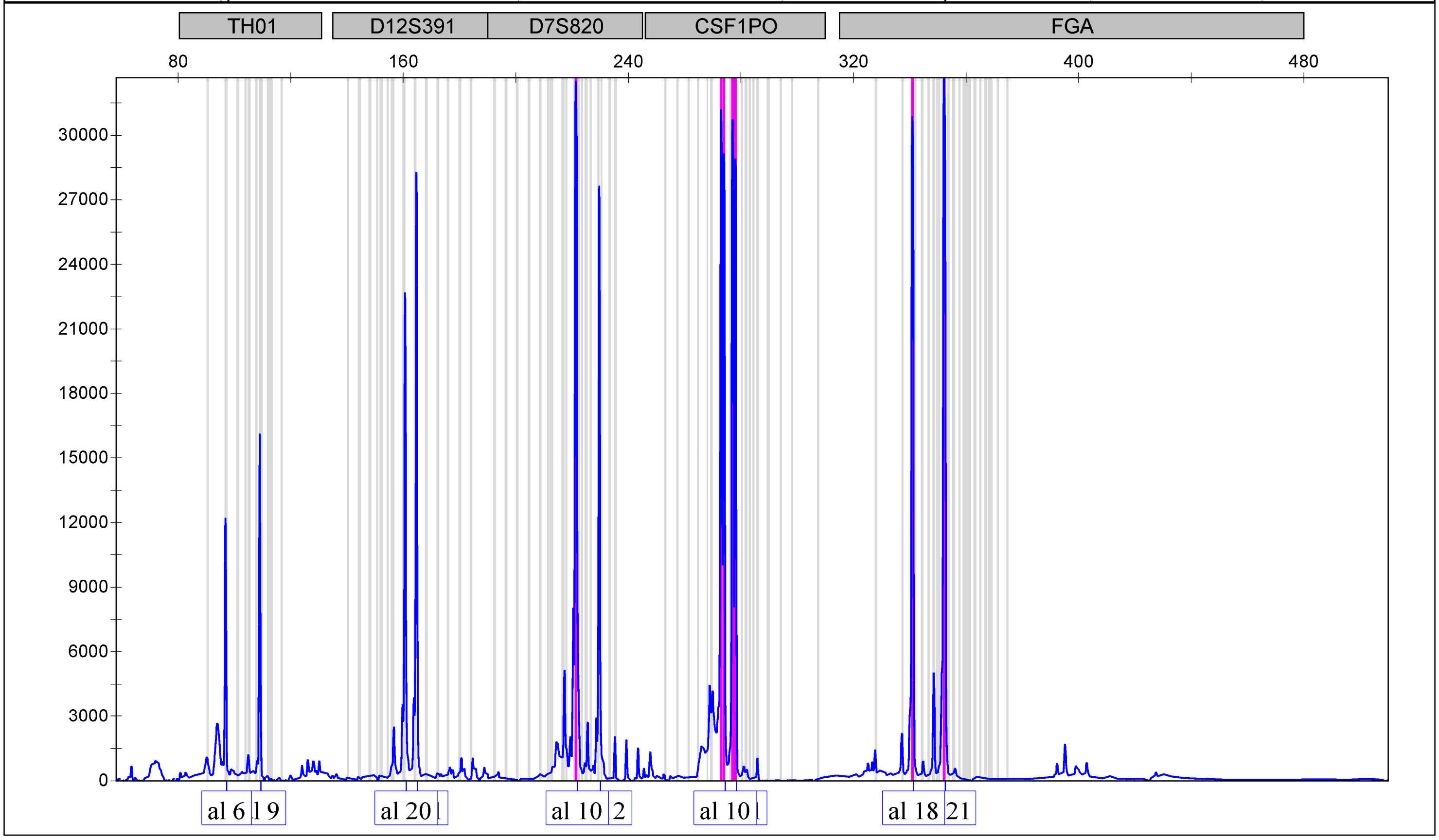

Sample File	Sample Name	Panel	OS	SQ
10_B02_XiBaoJianDing0318-2-0320.fsa	XRH83	STR Profile 2-dup		

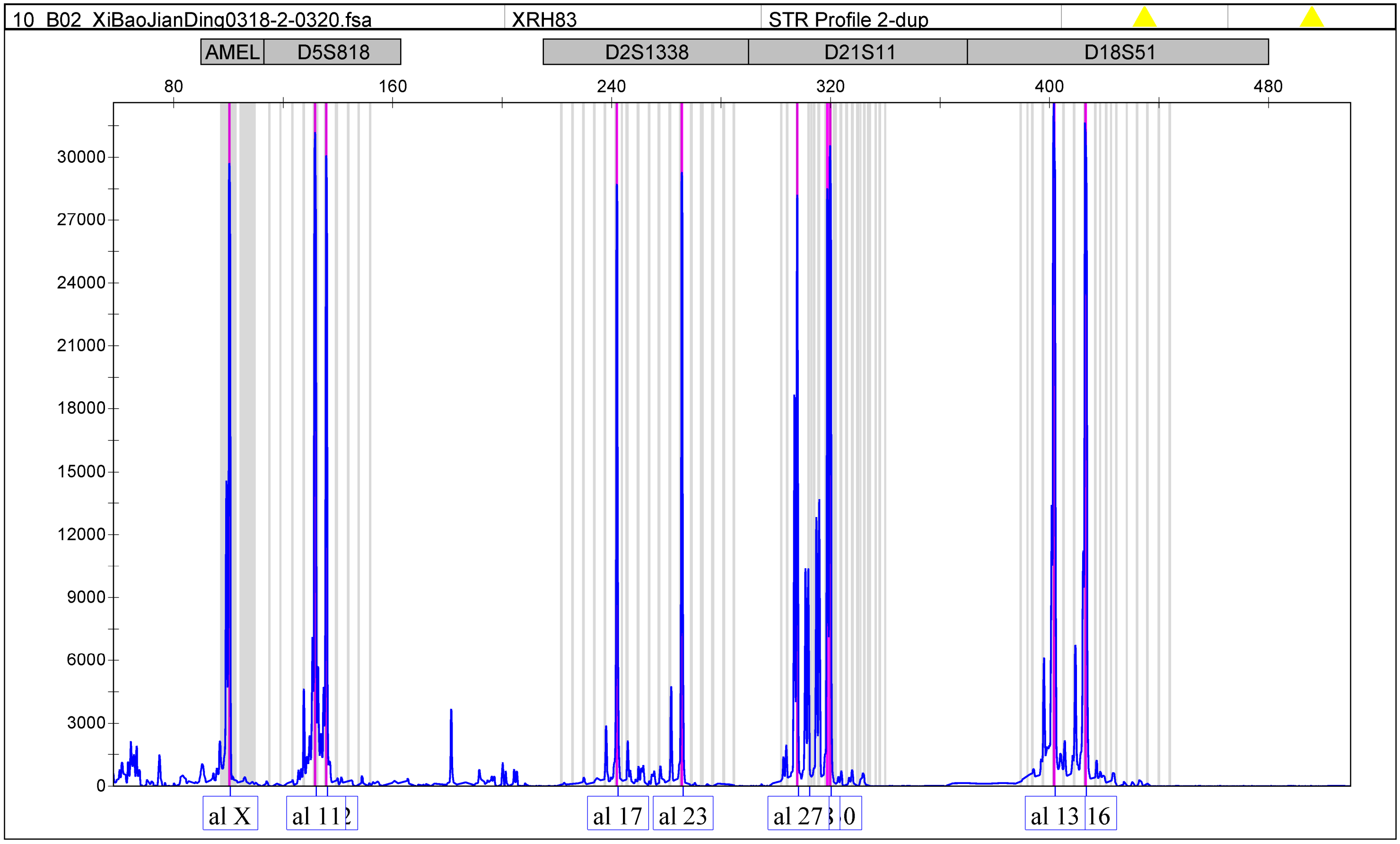

Sample File	Sample Name	Panel	OS	SQ
66_B09_XiBaoJianDing0318-2-0320.fsa	XRH83	STR Profile 3-dup		

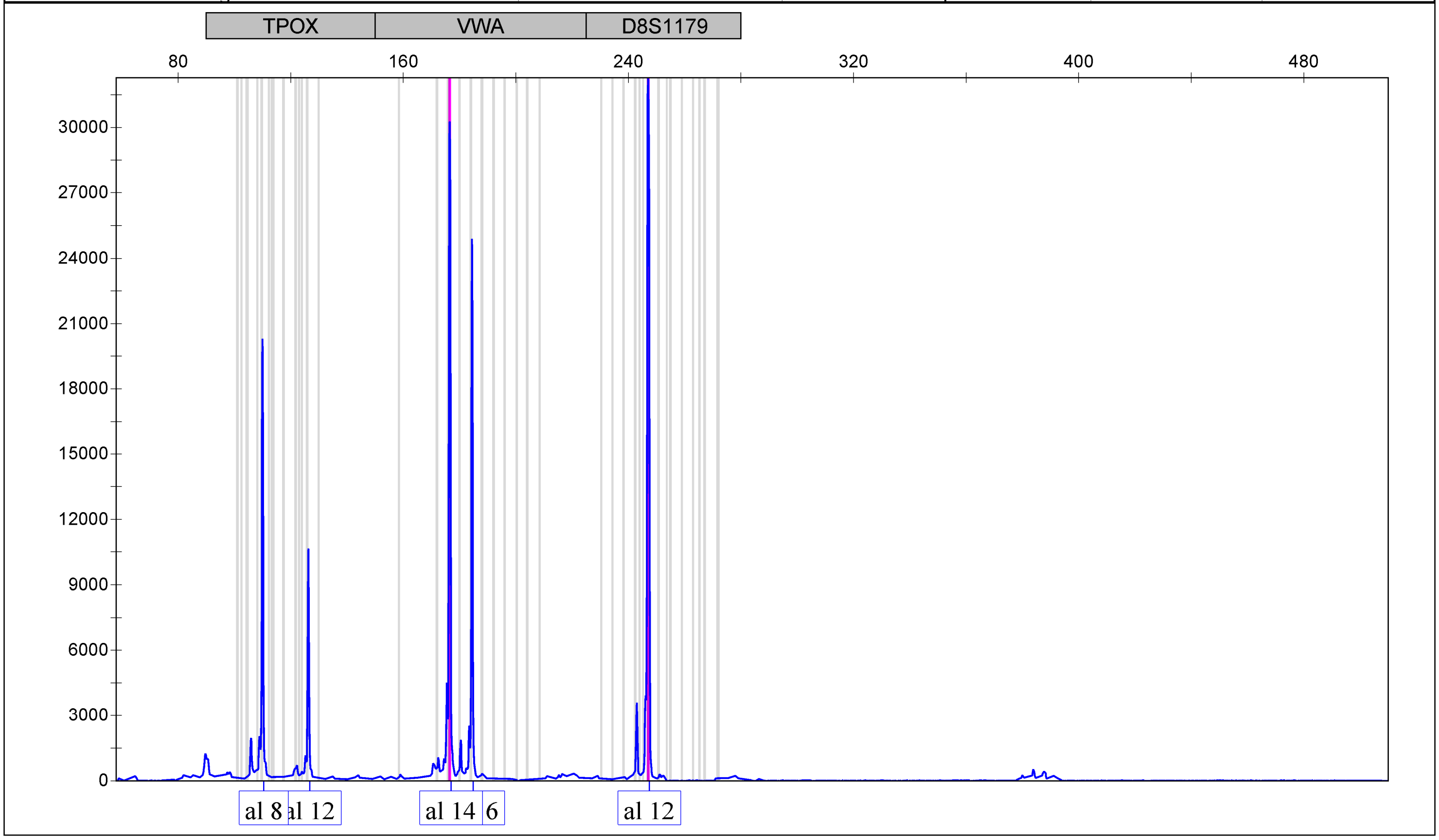

Sample File	Sample Name	Panel	OS	SQ
50_B07_XiBaoJianDing0318-3-0324.fsa	XRH83	STR Profile 4-dup		

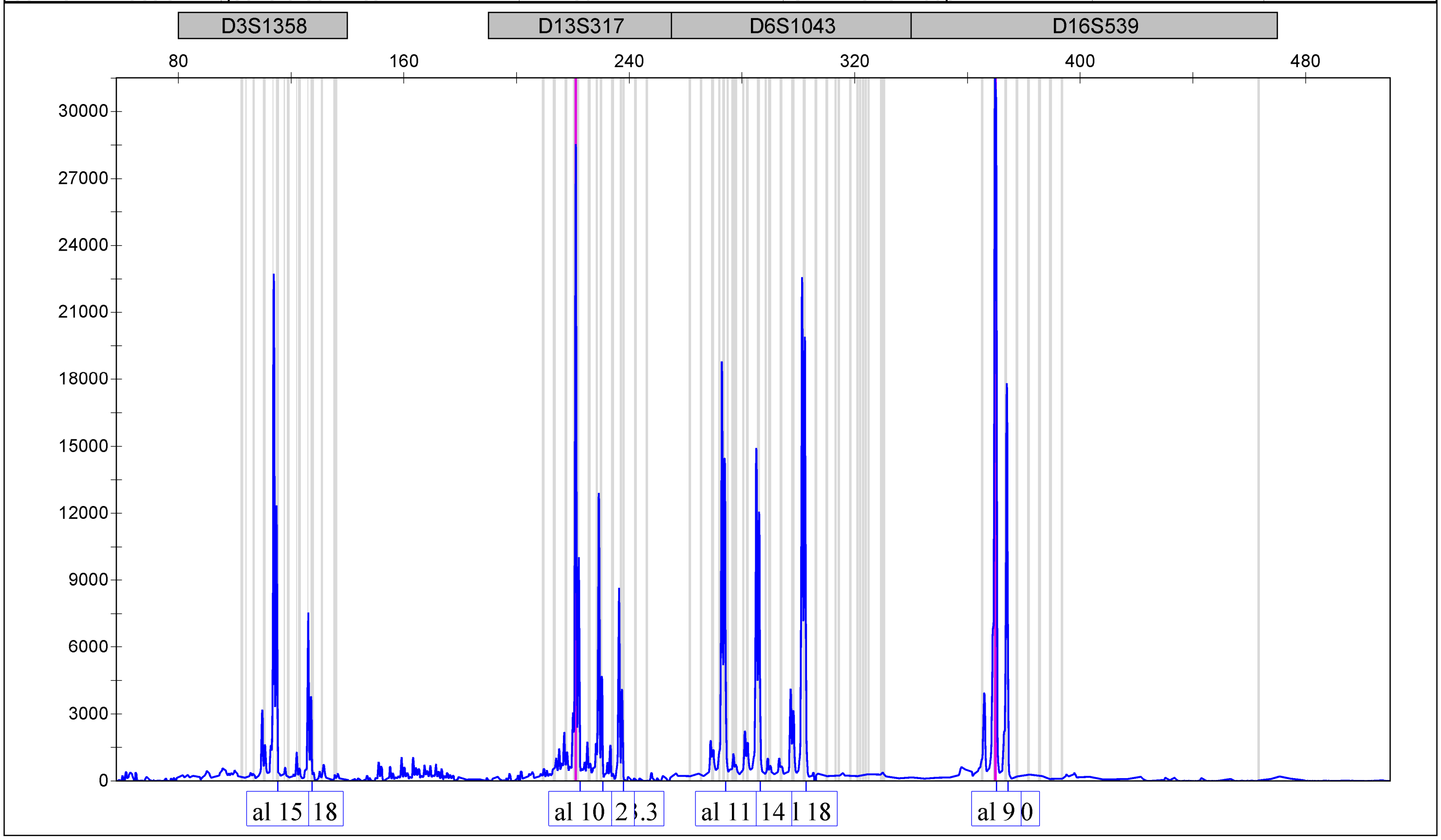

Applied Biosystems

GeneMapper 4.0

xibao-201503

Sample File	Sample Name	Panel	OS	SQ
50_B07_XBJD1-9XJ-49-10WZCXS11-12-0324.fsa	XRH83	STR Profile 5-dup		

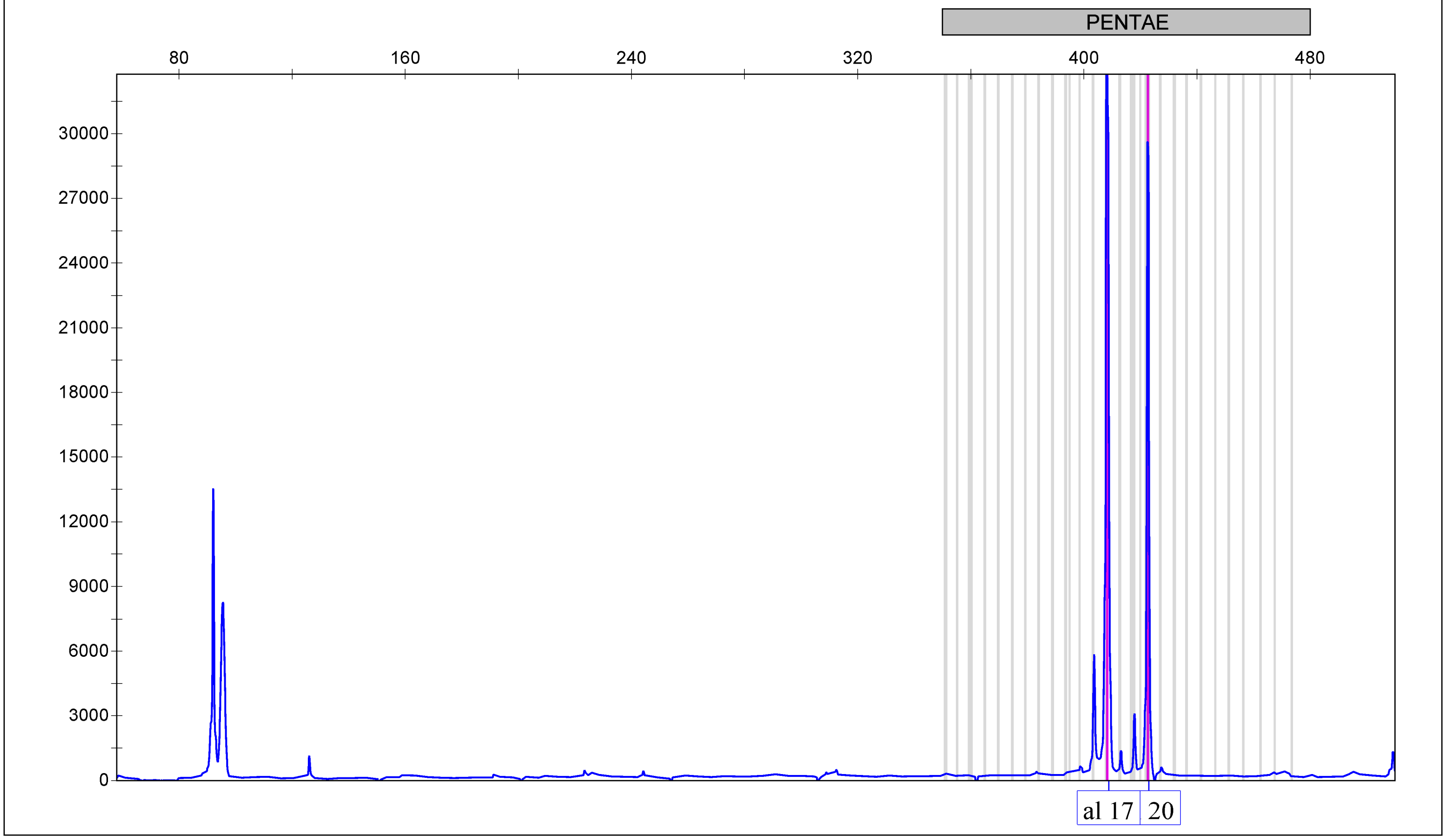

Sample File	Sample Name	Panel	OS	SQ
66_B09_XBJD1-9XJ-49-10WZCXS11-12-0324.fsa	XRH83	STR Profile 6-dup		

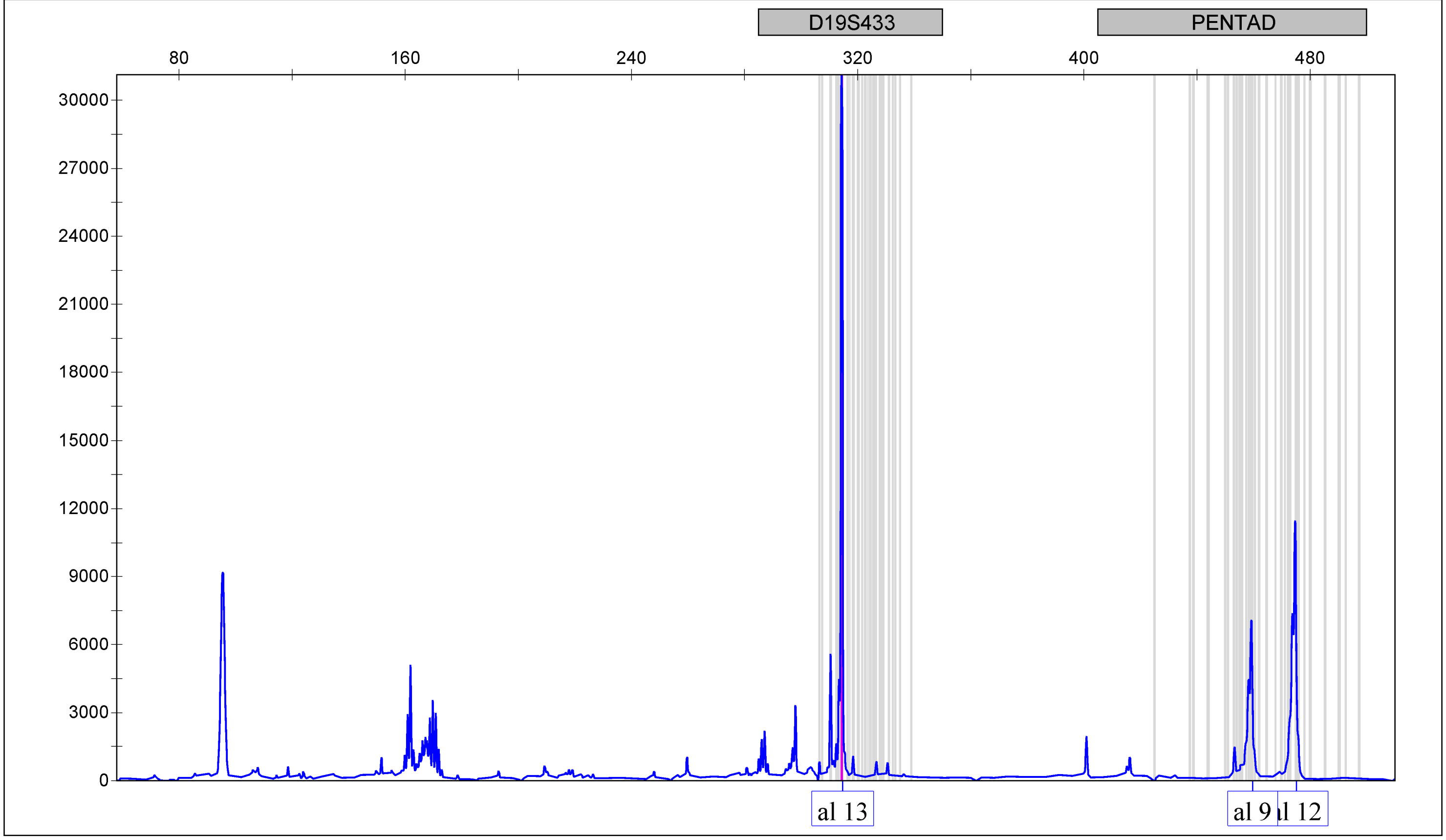

Supplement: Supplementary file 3 — SACC-83 cell line authentication [file 41419_2018_966_MOESM3_ESM.pdf]
