# Supplementary material for: The MYB/miR-130a/NDRG2 axis modulates tumor proliferation and metastatic potential in salivary adenoid cystic carcinoma
Source: Cell Death Dis. 2018 Sep 11;9(9):917. doi: 10.1038/s41419-018-0966-2 (PMC6134089; doi:10.1038/s41419-018-0966-2)
Supplement: Supplementary file 4 — Supplementary figure legends [file 41419_2018_966_MOESM4_ESM.docx]

**Supplementary Figure and Table Legends**

**Supplementary Table 1**: Correlations between NDRG2 expression and clinicopathologic parameters of adenoid cystic carcinoma.

**Supplementary Table 2**: the brand and product number of primary antibodies

**Supplementary Table 3**: Sequences of mRNA primers, miRNA mimics, inhibitors and siRNAs.

**Supplementary Figure 1:** Venn diagrams exhibiting that 2657 miRNAs were total mapped in the two groups, of which 176 miRNAs were dysregulated. Combined with an online database analysis, three candidate miRNAs regulating NDRG2 were identified.

**Supplementary Figure 2:** the relative miR-130a levels in miR-130a over-expression cells and the control cells.

**Supplementary Figure 3:** the relative mRNA levels of NDRG2 in miR-130a over-expression cells and the control cells.

**Supplementary Figure 4:** the reduction of NDRG2 protein amounts in miR-130a-overexpressing cell lines.

**Supplementary Figure 5:** the relative expressions of MYB in tumors and the corresponding normal salivary gland of fresh samples.
